# Alternating Modulation of Subthalamic Nucleus Beta Oscillations during Stepping

**DOI:** 10.1523/JNEUROSCI.3596-17.2018

**Published:** 2018-05-30

**Authors:** Petra Fischer, Chiung Chu Chen, Ya-Ju Chang, Chien-Hung Yeh, Alek Pogosyan, Damian M. Herz, Binith Cheeran, Alexander L. Green, Tipu Z. Aziz, Jonathan Hyam, Simon Little, Thomas Foltynie, Patricia Limousin, Ludvic Zrinzo, Harutomo Hasegawa, Michael Samuel, Keyoumars Ashkan, Peter Brown, Huiling Tan

**Affiliations:** ^1^Medical Research Council Brain Network Dynamics Unit at the University of Oxford, Oxford OX1 3TH, United Kingdom,; ^2^Nuffield Department of Clinical Neurosciences, John Radcliffe Hospital, University of Oxford, Oxford OX3 9DU, United Kingdom,; ^3^Division of Movement Disorders, Department of Neurology, Chang Gung Memorial Hospital, 333 Linkou, Taipei, Taiwan,; ^4^Department of Physical Therapy, Graduate Institute of Rehabilitation Science, Chang Gung University, 333 Taipei, Taiwan,; ^5^Unit of Functional Neurosurgery, Sobell Department of Motor Neuroscience and Movement Disorders, University College London Institute of Neurology, London WC1N 3BG, United Kingdom, and; ^6^Departments of Neurology and Neurosurgery, King's College Hospital, King's College London, London SE5 9RS, United Kingdom

**Keywords:** auditory cueing, beta modulation, freezing of gait, Parkinson's disease, stepping-related STN activity

## Abstract

Gait disturbances in Parkinson's disease are commonly refractory to current treatment options and majorly impair patient's quality of life. Auditory cues facilitate gait and prevent motor blocks. We investigated how neural dynamics in the human subthalamic nucleus of Parkinsons's disease patients (14 male, 2 female) vary during stepping and whether rhythmic auditory cues enhance the observed modulation. Oscillations in the beta band were suppressed after ipsilateral heel strikes, when the contralateral foot had to be raised, and reappeared after contralateral heel strikes, when the contralateral foot rested on the floor. The timing of this 20–30 Hz beta modulation was clearly distinct between the left and right subthalamic nucleus, and was alternating within each stepping cycle. This modulation was similar, whether stepping movements were made while sitting, standing, or during gait, confirming the utility of the stepping in place paradigm. During stepping in place, beta modulation increased with auditory cues that assisted patients in timing their steps more regularly. Our results suggest a link between the degree of power modulation within high beta frequency bands and stepping performance. These findings raise the possibility that alternating deep brain stimulation patterns may be superior to constant stimulation for improving parkinsonian gait.

**SIGNIFICANCE STATEMENT** Gait disturbances in Parkinson's disease majorly reduce patients' quality of life and are often refractory to current treatment options. We investigated how neural activity in the subthalamic nucleus of patients who received deep brain stimulation surgery covaries with the stepping cycle. 20–30 Hz beta activity was modulated relative to each step, alternating between the left and right STN. The stepping performance of patients improved when auditory cues were provided, which went along with enhanced beta modulation. This raises the possibility that alternating stimulation patterns may also enhance beta modulation and may be more beneficial for gait control than continuous stimulation, which needs to be tested in future studies.

## Introduction

Gait disturbances are an early sign and prominent feature of Parkinson's disease (Baltadjieva et al., 2006). In advanced stages many patients suffer from motor blocks (so-called freezing), festination, or balance problems (Ebersbach et al., 2013), which suggests that the basal ganglia contribute to the control of human gait. Gait disturbances are a major clinical challenge because they may be refractory to medication or deep brain stimulation (DBS) of basal ganglia targets, and drastically reduce patients' quality of life (Heremans et al., 2013; Fasano et al., 2015). Continuous DBS at a fixed high frequency, the current standard, may at some point be replaced by temporally flexible stimulation strategies (Arlotti et al., 2016; Meidahl et al., 2017), which increases the importance of understanding the neuronal population dynamics associated with gait control. Past studies recording EEG or subthalamic nucleus (STN) local field potentials (LFPs) have shown abnormally exaggerated neuronal synchronization when gait deteriorates (Singh et al., 2013; Shine et al., 2014), but they have not disclosed how basal ganglia activity is modulated during gait. To demonstrate modulation within the gait cycle, neural activity must be recorded with sufficiently high temporal precision. Direct LFP recordings from the STN in patients who have undergone deep brain stimulation surgery to alleviate motor symptoms provide this necessary high temporal resolution, and have helped to establish features of STN activity that correlate with upper limb movements in the past (Anzak et al., 2012; Tan et al., 2016a). In particular, beta oscillations (over ∼15–30 Hz) have been shown to be modulated during rhythmic movements of the contralateral upper limb with a beta trough during movement followed by a rebound between two consecutive movements (Androulidakis et al., 2008; Joundi et al., 2013).

Here we test the primary hypothesis that beta activity in the STN is also modulated during the stepping cycle. We sought to find out whether beta modulation is time-locked to the movement of the contralateral leg or synchronous in both STN. Because stepping involves coordinated and rhythmic movements of both lower limbs, we would expect beta modulation (relative power attenuation followed by a rebound) to alternate in an opposite manner between the two STN if it is locked to the contralateral step. EEG recordings during gait in healthy subjects also support this idea as beta oscillations from motor cortex seem to be modulated relative to the contralateral leg (Cheron et al., 2012; Bradford et al., 2016).

**Figure 1.**
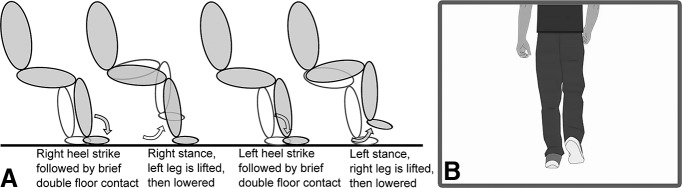
Schematic sequence of stepping in place and visual stimulus. ***A***, After each heel strike, the contralateral leg was lifted. The shaded limb depicts the right leg and the unshaded one the left leg. ***B***, One example picture from the video that dictated the stepping rhythm.

To assure patients' safety and to reduce movement artifacts, we primarily recorded STN LFPs during visually-guided stepping in place while sitting (Fig. 1). We asked patients to synchronize their steps with the rhythm of a walking cartoon man displayed in a video. This provided us with a measurement of step-timing accuracy. Our primary aim was to determine whether stepping was accompanied by rhythmic modulation of beta activity in the STN, and as such patients were recorded on medication to approximate normal stepping control as best as possible. Volitional stepping while sitting and free walking differ in that the latter requires balance adjustments, postural control, and arguably less attentional demand compared with that required for consciously controlled alternating limb movements. To confirm that results from our core paradigm were generalizable across conditions and applicable to real gait, we additionally recorded three patients during sitting and standing, and three patients during free walking.

Auditory cues should improve stepping performance, such as stepping accuracy, considering that auditory cues improve gait impairments in patients with Parkinson's disease (Hausdorff et al., 2007; Arias and Cudeiro, 2008, 2010; Mazilu et al., 2015). Auditory cues alone modulate sensorimotor beta oscillations even in the absence of movement (McIntosh et al., 1997; Iversen et al., 2009; Saleh et al., 2010; Fujioka et al., 2012, 2015) and the post-movement beta peak is more pronounced when feedback suggests that a movement was performed correctly (Tan et al., 2014, 2016b). Our second hypothesis was therefore that auditory cues would not only be associated with behavioral benefits in our paradigm, but also with enhanced beta modulation.

## Materials and Methods

### 

#### Participants

##### UK cohort.

We recorded 16 Parkinson's disease patients who had undergone bilateral implantation of DBS electrodes in the STN. The study was approved by the local ethics committee and patients gave informed written consent before the recording.

Three full datasets and one electrode that was located on the right had to be excluded because of severe movement artifacts (see exclusion criteria in the section “Data Processing”). Clinical details of the patients included [age 61 ± 4 years (mean ± SD), mean disease duration 12 ± 4 years, 1 left-handed, 1 female] are listed in Table 1. Levodopa equivalent dose was calculated according to Tomlinson et al. (2010). Patients took their prescribed dopaminergic medication as usual and recordings were performed 3–7 d after the surgery at one of the following three surgical sites: King's College hospital in London, University College hospital in London, or the John Radcliffe hospital in Oxford, UK. For each patient, one of the following three macroelectrode models was used: Medtronic 3389 (quadripolar, *n* = 8), Boston Scientific DB-2201 Vercise (octopolar, *n* = 3), and Boston Scientific DB-2202 Vercise directional (octopolar, directional, *n* = 2).

**Table 1.** Clinical details of all patients included

ID	Age, years/sex/dom hand	UPDRS-III OFF/ON levodopa	Disease duration, years	Main symptom	Levodopa equivalent dose, mg/d	DBS lead
1	62/M/R	27/4	12	FOG	955	Medtronic 3389
2	60/M/R	52/30	8	FOG	1282	Medtronic 3389
3*	59/M/R	53/18	7	Tremor, bradykinesia, dyskinesia	1195	Boston Scientific DB-2202
4*	64/F/R	66/36	16	Rigidity, tremor, FOG	1628	Boston Scientific DB-2202
5*	59/M/R	36/8	14	Fluctuations, tremor	1062	Medtronic 3389
6*	56/M/L	42/26	7	Fluctuations, dyskinesia	1365	Medtronic 3389
7*	62/M/R	59/15	12	Tremor, rigidity, dyskinesia, mild FOG	1000	Medtronic 3389
8*	71/M/R	36/18	15	Tremor, FOG	785	Boston Scientific DB-2201
9*	61/M/R	33/11	9	Rigidity	1293	Medtronic 3389
10*	57M/R	49/18	12	Tremor, FOG	1881	Boston Scientific DB-2201
11*+	59/M/R	28/8	10	Tremor, mild FOG	1010	Boston Scientific DB-2201
12+	59/M/R	23/6	22	Fluctuations, mild FOG	1658	Medtronic 3389
13+	65/M/R	16/8	8	Tremor	1434	Medtronic 3389
14FW	69/F/R	49/8	20	Tremor, fluctuations and FOG	1600	Medtronic 3389
15FW	64/M/R	44/7	12	Bradykinesia, fluctuations and gait	2391	Medtronic 3389
16FW	68/M/R	33/22	13	Bradykinesia, Rigidity and FOG	1830	Medtronic 3389

*, Recorded in both the soundOff and soundOn conditions during sitting; +, recorded during stepping while standing (soundOff only); FW, recorded during free walking in Taiwan; Dom hand, dominant hand; R, right; L, left; UPDRS-III, Unified Parkinson's disease rating scale part III OFF levodopa/ON levodopa (obtained not at the time of the recording but preoperatively).

Levodopa equivalent dose was calculated according to Tomlinson et al. (2010).

##### Taiwan cohort.

In the Chang Gung Memorial Hospital in Taiwan, three Parkinson's disease patients, who received STN DBS surgery (using the Medtronic 3389 macroelectrode model), were recorded 5 d after the surgery during ∼4 m of straight walking. Walking was uncued, i.e., no video was presented. Patients were recorded after overnight withdrawal of dopaminergic medication. Trunk acceleration was recorded with a triaxial accelerometer fixed with tape over their lumbar spine. The procedure was approved by the local ethics committee.

#### Task

In the stepping paradigm, patients were seated in a chair in front of a laptop and two pressure sensors were placed on the floor such that their feet could comfortably reach the plates (Scythe, USB 3FS-2 foot pedal or Biometrics ForcePlates). The laptop displayed a video of a walking cartoon man (see Fig. 1), which was looped after one walk cycle (i.e., 1 right and left step, separated by 1 s) such that the man was walking in place. Patients were instructed to step onto the leftmost and rightmost plate with their left and right foot, respectively, in synchrony with the footsteps of the man in the video while resting their arms on their lap. They were asked to synchronize their steps with the steps in the video as precisely as possible. The contralateral foot was lifted shortly after each heel strike, resulting in a brief period of double support, which also exists during upright walking (Hollman et al., 2011).

In one condition, patients were stepping while sitting and had to rely on the video only (soundOff). In a second condition (soundOn), a metronome sound was provided at the time of each heel strike displayed in the video and thus could provide additional information about the timing of heel strikes via the auditory system. Nevertheless, patients were still asked to synchronize their steps with the steps in the video and no further instruction was given regarding the auditory cues. For a control condition, patients were instructed to watch the video without moving and to think of anything unrelated to walking, and were provided with specific thought examples such as past holidays or upcoming plans. The duration of the video shown in each run was 42 s and thus contained 21 left and 21 right heel strikes. After a short practice run, we recorded the three conditions [NS = SoundOff + movement, S = SoundOn + movement, C = Control condition (watching only)] in the following order:

##### 2x NS, 2x S, 3x C, 2x S, 2x NS.

This order was chosen such that one stepping in place condition without sound (NS) preceded the condition with sound (S) to make sure patients performed one stepping in place condition without prior exposure to the sound. The conditions were ordered such that temporally linear drifts over time would be factored out. The soundOff condition was always the first condition we recorded to avoid any initial carryover effects of the auditory rhythm. Ten patients were recorded in both sound conditions, the remaining four were only recorded without the additional soundOn condition. Three patients were also recorded during stepping in place while standing to evaluate whether the activity changes found in the seated task resemble the changes during stepping while standing. To make sure patients could not fall or move too far away from the amplifier, an experimenter stood closeby. All conditions that required stepping movements were recorded four times, but the control condition was only recorded three times to account for the fact that movement-related artifacts occasionally resulted in exclusion of steps in the former.

After each of the “watching only” (C) runs, subjects rated their performance with a questionnaire asking “How well were you able to think of something else rather than walking?” for the control condition (C). Patients indicated their rating on a visual analog scale ranging from 0 to 10 with 10 corresponding to “Very well” and 0 to “Not at all”.

After the recording was completed, patients filled in the *freezing of gait questionnaire* to assess the presence of preoperative gait problems (Giladi et al., 2000).

In the free-walking paradigm, the patients (from the Taiwan cohort) were asked to walk along a ∼4 m straight line backward and forward at their own comfortable pace.

#### Recordings

A TMSi Porti amplifier (2048 Hz sampling rate, common average reference; TMS International) was used to record monopolar LFPs, the timing of the footsteps via the pressure sensors, the triggers for the heel strikes displayed in the video, and the accelerometer output (data available on request). The data recorded in Taiwan did not contain pressure sensor input or triggers for the video because no video was presented. The triaxial accelerometer used in Taiwan was a TMSi 3D accelerometer (TMS International).

The triggers were registered with a light-sensitive sensor attached to the top left corner of the presentation laptop. In the video underneath the light-sensitive sensor, a black square turned white for one frame (41.7 ms at a frame rate of 24 frames/s) with each right heel strike, and gray [RGB = (179, 179, 179)] with each left heel strike. This was completely covered by the sensor and thus not visible to the patients.

#### Data processing

All analyses were performed in MATLAB (v2016a, MathWorks; RRID:SCR_001622). The data were re-referenced off-line to obtain more spatially focal bipolar signals by subtracting the data from neighboring electrode contacts. If single channels were saturated or inactive, the remaining surrounding contacts were subtracted instead. This was the case for three octopolar electrodes (Boston Scientific, DB-2201 and DB-2202) of which 1 + 2 + 4 neighboring channels were flat. The triggers of the first two heel strikes and of the last one were deleted to exclude start and stop-related activity. Trials with movement artifacts were discarded following visual inspection and bipolar channels that were strongly contaminated with artifacts resulting from cable movement during stepping were excluded. Thus we included only bipolar combinations, where the SD of the 1 Hz high-pass and 40 Hz low-pass filtered event-related potential (−0.5:1.5 s locked to the stepping movement) did not exceed 1.5 μV (the average SD of the included channels was 0.40 ± 0.31 μV). This way, impact-related artifacts that were locked to the movement were excluded; however, some channels were strongly contaminated by broadband artifacts that were not precisely locked to the movement and were thus not visible in the event-related potential. To classify impact-related broadband artifacts we examined the time-frequency decomposed signal (using the same parameters as described in the section “Time-frequency decomposition”) and removed those bipolar channels in which a broadband power peak was observed within a 0.2 s window in frequencies up to 25 Hz. This resulted in an average of six bipolar channels that were included per patient.

Activity for each STN was computed by averaging across all bipolar channels of the electrode to avoid any channel selection bias in the sitting condition. This is a conservative approach as not all contact pairs are likely to have been in the STN. Our results would remain the same when only the contact pairs with the highest 20–30 Hz modulation were chosen. Sixty-eight percent of all bipolar contacts with the highest beta modulation from each lead included at least one contact used for therapeutic stimulation and 90% included at least one of the therapeutic contacts or one that is directly adjacent. Inspection of the postoperative CT or MR scans confirmed that for 91% of these bipolar contacts at least one of the two contacts was consistent with location in the STN.

For the average spectra and the statistics in the standing and free walking condition, we included only the channels with the highest 20–30 Hz modulation to increase statistical power, given the small sample size.

#### Behavioral analyses

**Figure 2.**
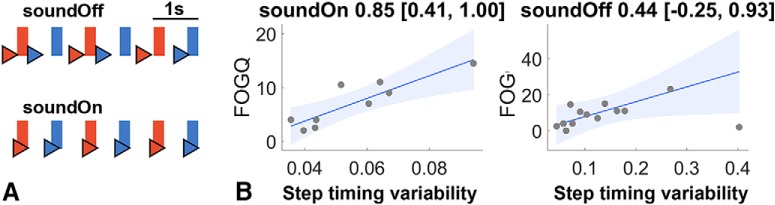
***A***, Example of the timings of the heel strikes (triangles) relative to the cues (rectangles) presented in the video of one participant (P5; red, right heel strike; blue, left heel strike). We call the difference between rectangles and the corresponding heel strike step-to-cue difference and the variability of these differences step timing variability. When the sound was off (top row), heel strikes were less rhythmic and less well synchronized with the video than when it was on (bottom row), which was expressed as a larger step timing variability. ***B***, Correlation between step timing variability and the FOG-Q scores. Correlations with the behavioral data from the soundOn condition are to the left (*n* = 9) and correlations with the data from the soundOff condition are to the right (*n* = 13). The titles show Spearman's ρ with the 95% bootstrapped CIs. The line denotes the robust linear regression fit with 95% CIs.

The two main behavioral variables of interest were step-to-cue difference and step interval durations. *Step-to-cue differences* denote the difference between each real step and the closest corresponding heel strike displayed in the video (Fig. 2*A*). *Step interval durations* denote the length of the interval between the current and the consecutive (contralateral) step registered by the foot pedal. If patients found it hard to step on time with the cue, it resulted in a larger variability of step-to-cue differences, which could be negative and positive and could thus nevertheless result in an average step-to-cue difference close to zero. To quantify step timing variability, we thus computed the median absolute deviation of all step-to-cue differences, which is a robust measure of variability (Williams, 2011). We also computed the median absolute deviation of all step interval durations, which we will call step interval variability.

Step-to-cue differences longer than 1 s and step interval durations longer than 2 s were discarded as outliers [mean number of outliers = 10 ± 9.9 (SD)]. This resulted in an average number of 51 ± 30 left and 49 ± 30 right steps in the soundOff condition and 44 ± 20 left and 43 ± 17 right steps in the soundOn condition. Then the median of the remaining step-to-cue differences and step interval durations was computed. One patient started with the wrong foot and was stepping with the opposite foot to the one shown in the video in some runs. Those steps were excluded for the correlation analyses as these step-to-cue differences could arbitrarily be interpreted as close to −1 or 1.

#### Time-frequency decomposition

The data were downsampled to 1000 Hz and high-pass filtered (1 Hz cutoff, Butterworth filter, filter order = 6, passed forward and backward) before applying continuous Morlet wavelet transforms using the *fieldtrip*-function *ft_freqanalysis* (RRID:SCR_004849; Oostenveld et al., 2011). The wavelets were set to span 6 cycles for frequencies between 5 and 45 Hz and to span 12 cycles for frequencies between 55 and 90 Hz. The resulting time-frequency decomposition was downsampled to 200 Hz and smoothed by averaging within a 0.2 s sliding window to reduce noise in the data, which aids in performing cluster-based permutation statistics. Relative power was obtained for each subject and frequency by normalizing the absolute power by its average across time for each channel: (*power* − *average power*)/*average power* × *100*.

To compare the degree of the left/right-alternating modulation difference between the low- and high-beta band, we quantified this as median squared power difference between the relative power from the right and left STN [median (*power*_riSTN_ − *power*_leSTN_)^2^].

#### Statistics

Behavioral variables or relative power changes are reported as mean ± SD. For the soundOn versus soundOff comparison, the data from both STN aligned to the contralateral heel strike were averaged to avoid overinflation of the sample size. This resulted in a sample size of 10 subjects for these comparisons.

Multiple-comparison correction for power across multiple time and frequency bins was performed by using a cluster-based permutation correction approach (Maris and Oostenveld, 2007): the condition labels of the original samples were randomly permuted 2000 times such that each data pair was maintained but its order of subtraction might change to create a null-hypothesis distribution. If relative power was tested for significant differences from zero, then the sign changed from “+*relative power*” to “−*relative power*” if a data point was permuted. Suprathreshold clusters (pre-cluster threshold: *p* < 0.05) were obtained for the original unpermuted data and for each permutation sample by computing the *z*-scores relative to the permutation distribution. If the sum of the *z-*scores within the original suprathreshold-clusters exceeded the 95th percentile of the 2000 largest sums of *z-*scores from the permutation distribution, it was considered statistically significant.

Data for pairwise comparisons were first subjected to Lilliefors tests to assess normality, and then to two-tailed *t* tests (if normality was given) or to Wilcoxon signed-rank tests (if the normality assumption was violated). Differences were considered significant if *p* < 0.05 and the corresponding test-statistics and exact *p* values are reported. Effect sizes are reported as *Hedges g* and its 95% bootstrapped CIs, which were estimated with the MATLAB *Measures of Effect Size Toolbox* (Hentschke and Stüttgen, 2011). To control for multiple comparisons of several beta burst properties, the false discovery rate correction procedure was performed (Benjamini and Hochberg, 1995).

#### Correlation analyses

Correlations were computed as Spearman's rank correlation coefficients using the *Spearman* function from the Robust correlation toolbox, which also provides bootstrapped confidence intervals (Pernet et al., 2013). Spearman's ρ is less affected by outliers than Pearson's *r* because it is based on the rank-transformed data. The latter can only be correctly computed based on the assumption that outliers are absent, which could not be assured. To visualize linear trends nevertheless, robust linear regression fits and their 95% CIs were computed using an iteratively reweighted least-squares algorithm (Holland and Welsch, 1977).

Correlations between STN power and the interval to the neighboring step were computed across trials (i.e., steps) within each subject, Fisher's *z*-transformed and then also subjected to the cluster-based permutation correction procedure to test whether the direction of these correlations was consistent across patients.

#### Beta burst properties

We also examined whether increased beta power, appearing in the trial-average, resulted either from a consistent amplitude increase of beta oscillations across trials or instead from an increased likelihood of these beta bursts in some trials. In the trial-average of the power these two possibilities cannot be distinguished. Beta bursts were classified as periods where the amplitude exceeded a threshold defined as the 75th percentile of the amplitude concatenated across conditions (Tinkhauser et al., 2017). The beta amplitude was filtered between 28 and 30 Hz according to the power difference in the soundOn-soundOff comparison (Butterworth filter, filter order = 6, passed forward and backward, and smoothed by a 0.3 s moving average to avoid misclassification of noise as bursts). The median amplitude and median duration of these bursts were computed within a 0:1 s window after the contralateral heel strike, averaged for each patient in the soundOn and soundOff condition, and then compared.

## Results

### Behavioral results while sitting and stepping in place

The average step-to-cue difference (*n* = 13, averaged across soundOff and soundOn if present) was −0.09 ± 0.1 s (SD), so that the patient's step was on average 100 ms earlier than the heel strike displayed in the video, and patients were able to anticipate the next step (*negative mean asynchrony*). The average step interval duration was 0.99 ± 0.03 s confirming that patients successfully matched their step intervals to the 1 s interval in the video.

When the sound was provided, a significant reduction in step timing variability relative to the soundOff condition was observed [soundOff = 0.13 ± 0.11 s, soundOn = 0.06 ± 0.02 s, Wilcoxon signed rank test (*n* = 9): *p* = 0.020, Hedges *g* = 0.85 (0.54, 1.85)]. We found no significant difference in step-to-cue differences [soundOff = −0.10 ± 0.09 s, soundOn = −0.13 ± 0.09 s, *t*_(8)_ = 1.0, *p* = 0.331, Hedges *g* = 0.29 (−0.27, 0.91)], step interval durations [soundOff = 0.97 ± 0.08 s, soundOn = 1.0 ± 0.01 s, Wilcoxon signed rank test (*n* = 9): *p* = 0.125, Hedges *g* = 0.57 (0.23, 1.26)] or step interval variability (soundOff = 0.09 ± 0.05 s, soundOn = 0.07 ± 0.04 s, *t*_(8)_ = 1.5, *p* = 0.171, Hedges *g* = 0.50 (−0.07, 1.47)]. Note though, that the 95% CIs of the effect size for the step interval duration comparison did not include zero, which indicates that step intervals were slightly longer when the sound was provided.

To synchronize with the heel strikes in the video separated by 1 s intervals, the timing of the next step had to be delayed if the current step was very early and it had to be advanced if the current step was late. Hence, step interval duration should be negatively correlated with the preceding step-to-cue difference. *t* Tests on the Fisher's *z*-transformed correlations of each subject showed that this was the case for both conditions (soundOff: ρ = −0.35 ± 0.23, *t*_(8)_ = −4.9, *p* = 0.001; soundOn: ρ = −0.55 ± 0.19, *t*_(9)_= −7.51, *p* < 0.001). Note that this correlation was stronger for the soundOn condition even though the variability of both variables was higher when no sound was provided. This indicates that auditory feedback facilitated step interval adjustments as evidenced by a stronger relationship between step-to-cue differences and the duration of the subsequent step interval.

Patients' self-reports suggested that they were successful in thinking of something unrelated to walking in the control condition, as the question “How well were you able to think of something else rather than walking?” resulted in 7.7 ± 1.8 points of 10 (corresponding to “Very well”).

### Correlation between task performance and gait questionnaire

We also examined whether step timing variability, the only performance measure that improved significantly with the sound, was worse in patients with more severe preoperative gait problems. Severity of gait problems was assessed with the freezing of gait questionnaire (FOG-Q; Giladi et al., 2000). The FOG-Q scores correlated with the step timing variability in the soundOn condition [Fig. 2*B*, left; ρ = 0.85 (0.41, 1.00), *p* = 0.004]. These clinical correlates of step timing variability support the use of the latter measure as a surrogate index relevant to gait performance in our paradigm. Only a weak trend was seen in the same direction in the soundOff condition [Fig. 2*B*, right; ρ = 0.44 (−0.27, 0.93), *p* = 0.137].

### Power modulation

**Figure 3.**
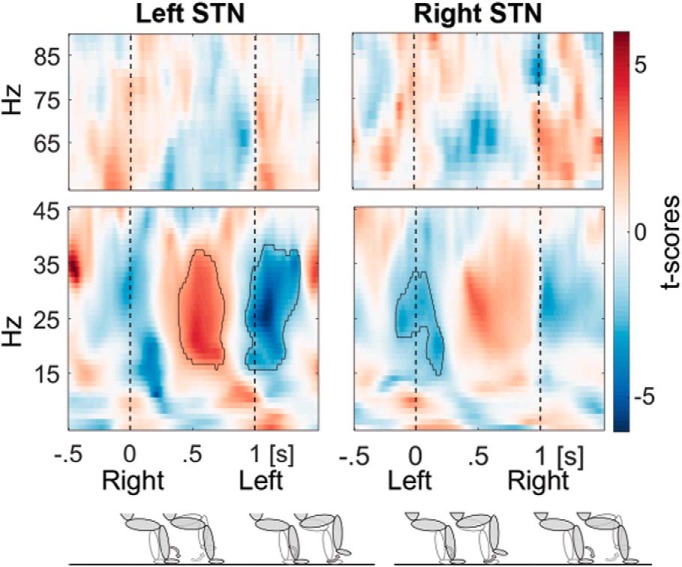
Beta and gamma step-related power modulation. Beta power was modulated in an opposite manner in the left (*n* = 13) and right STN (*n* = 12) during active stepping aligned to the contralateral heel strike (at 0 s; pooled across the soundOn and soundOff condition). The ipsilateral heel strike occurred on average 1 s afterward. The bottom row shows the concurrent leg movements and that shortly after each heel strike the contralateral leg is lifted for the next step. The shaded limb depicts the right leg and the unshaded one the left leg. Encircled clusters denote significant power increases according to the cluster-based permutation procedure in red and decreases in blue relative to the average within the step cycle.

**Figure 4.**
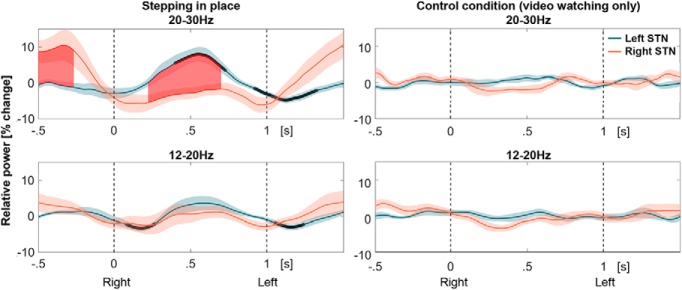
High- and low-beta power modulation. The data are aligned to all right heel strikes (at 0 s), followed by left heel strikes on average 1 s afterward. Black bold lines denote significant differences to 0 and the red filled area shows significant differences between 20 and 30 Hz beta power in the left and right STN after cluster-based multiple-comparison correction (top row; *n* = 12 patients). The significant differences between the left and right STN appeared when one foot is resting on the floor while the other is up in the air. Shaded areas denote SEM. No significant modulation was present when patients were not moving but were watching the video (right column).

Beta power was significantly modulated during stepping in place in both STN (Fig. 3; data averaged across the soundOn and soundOff conditions) with strong decreases at the time of the heel strikes when the opposite leg was lifted. Beta power was highest after the contralateral heel strike when the contralateral foot rested on the ground as shown by the red power increase in Figure 3. Note that the data of the left STN is aligned to the right heel strike, whereas the data of the right STN is aligned to the left heel strike, so that beta power in the two STN was modulated in an opposite manner (Fig. 4). Figure 3 also raises the possibility that gamma activity was modulated opposite to beta activity within each STN. However, cluster analysis failed to identify significant clusters in the gamma band and so changes in this frequency band were not considered further.

The step-specific modulation was particularly pronounced in the high-beta band (20–30 Hz) and less so in the low-beta band (12–20 Hz; Fig. 4). The degree of the left/right-alternating modulation difference was significantly higher in the high-beta band compared with the low-beta band during stepping in place [Wilcoxon signed rank test, *n* = 13, *p* = 0.021, Hedges' *g* = 0.60 (0.28, 1.07)], but not during video watching without movement [Wilcoxon signed rank test, *n* = 13, *p* = 0.733, Hedges' *g* = −0.35 (−0.87, 0.26)].

### Power modulation during stepping while standing resembles that during stepping while sitting

**Figure 5.**
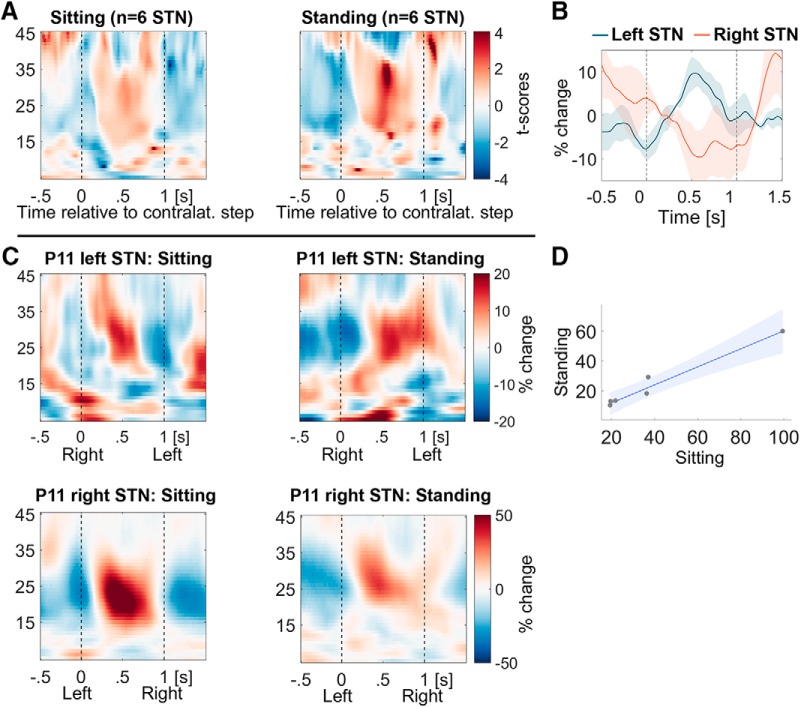
Power modulation during stepping while standing resembles the modulation during sitting. ***A***, The increase in beta power after the contralateral step during stepping while standing (right) looks very similar to the modulation while sitting (left; *n* = 6 STN; compare Fig. 3). ***B***, The 20–30 Hz power average (line plot) shows a similar alternating pattern to that in Figure 4. ***C***, Beta modulation in both hemispheres and both tasks from one example patient (P11). ***D***, The degree of modulation during stepping while standing closely correlates with the degree of modulation while sitting across the six recorded STN.

To confirm that the step-related activity we observed during sitting could be used to infer stepping activity when erect, we sought correspondence between stepping during sitting and standing in a small test cohort (6 STN; Fig. 5*A*,*B*). The 20–30 Hz modulation during standing and stepping on the spot also was significant [*t*_(5)_ = 3.0, *p* = 0.030, Hedges' *g* = 2.23 (1.68, 4.32)]. Modulation during stepping while standing correlated perfectly with that while sitting (ρ = 1.00 *p* < 0.001 in the 6 STN; Fig. 5*D*). Figure 5*C* shows the time-frequency spectra in both conditions for a direct visual comparison from one example patient.

### Power modulation during free walking resembles that during stepping

**Figure 6.**
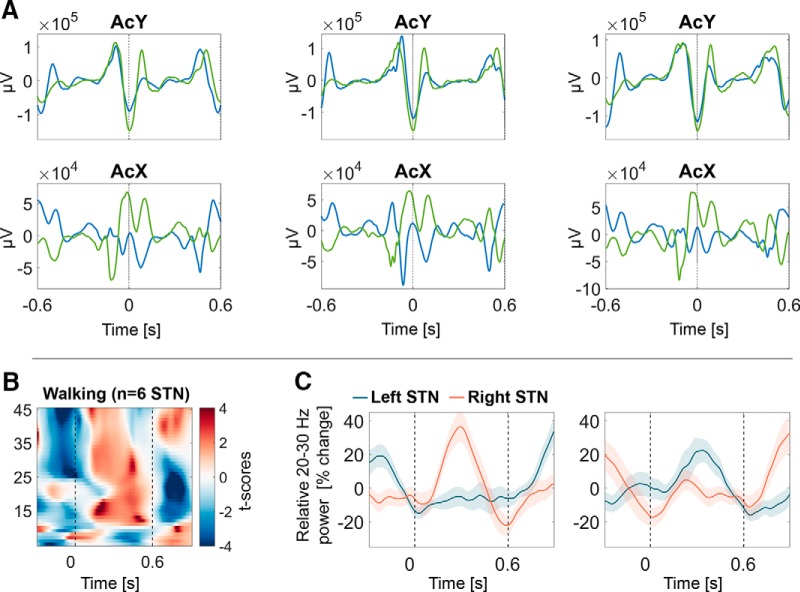
During straight unrestrained walking, beta power was modulated in an opposite pattern, similar to what was observed during stepping while sitting and standing. ***A***, Peaks in the accelerometer (*y*-axis) were classified as heel strikes. Every other step was chosen in each sequence of consecutive steps, and the uniformity of unilateral heel strike was confirmed by examination of the signal in the *x*-axis of the accelerometer which captured left-right movement. ***B***, Average beta modulation from three patients (*n* = 6 STN) during walking, which resembles Figure 5*A*. ***C***, 20–30 Hz beta modulation from one example patient when aligned to either even or odd steps.

Stepping in place while sitting is a task that all patients could accomplish and that avoids any risk of falling. However, to further corroborate the value of inferences made from data recorded during stepping while sitting we checked to see whether similar patterns of beta modulation were evident in a small selected cohort of patients during gait. Note, though, that these patients were tested off medication. Their step intervals during free walking were shorter (average step interval duration 0.61 ± 0.01 s) compared with the 1 s interval during cued stepping but not more variable (step interval variability 0.05 ± 0.01). On average 45 ± 16.5 steps were included after heel strikes were detected according to peaks in the *y*-axis (up-down) of the accelerometer recordings. Every other step was chosen in each sequence of consecutive steps, and the uniformity of unilateral heel strike was confirmed by examination of the signal in the *x*-axis of the accelerometer which captured left-right movement (Fig. 6*A*).

The average time-frequency spectra of the six STN recorded from three patients exhibited very similar modulation (Fig. 6*B*) to the spectra during stepping while sitting or standing in Figure 5*A*.

### Modulation increased with auditory cueing

**Figure 7.**
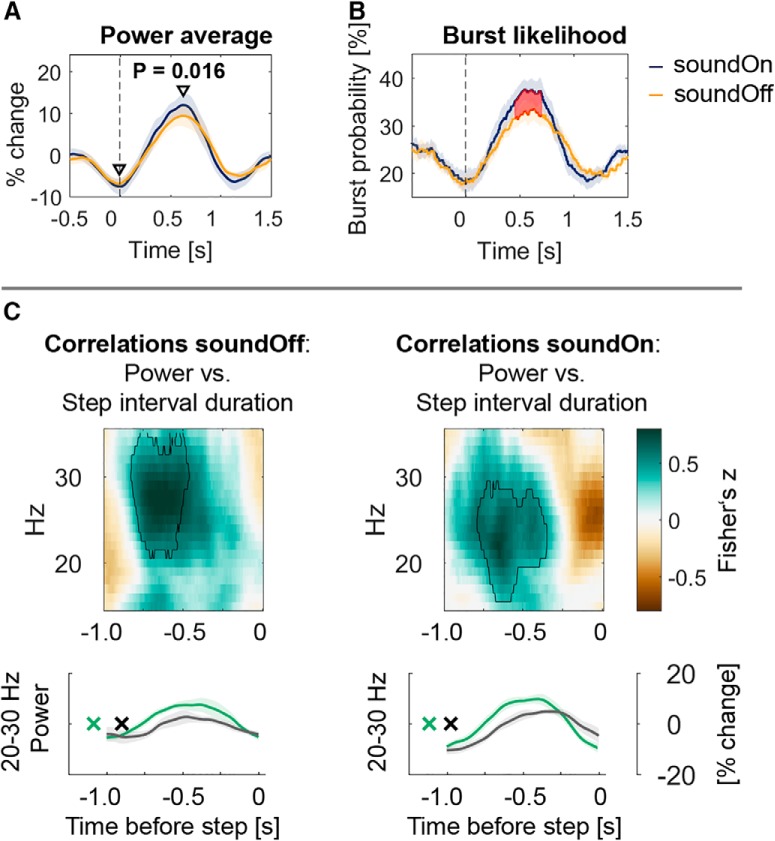
Beta bursts after contralateral heel strikes (at time = 0 s) were more likely when auditory cues were provided. ***A***, Power modulation [*max-min(power*)] was significantly higher between 28 and 30 Hz during stepping with the metronome compared with stepping without the sound and this difference was mainly driven by an increased beta power peak after the contralateral step (*n* = 9 patients; *t*_(8)_ = 3. 05, *p* = 0.016). ***B***, 28–30 Hz beta bursts (defined as exceeding the 75th percentile of the amplitude) were more likely to occur when the sound was on. This probability mirrors the power average to the left. ***C***, Correlations between STN power and step interval duration. Fisher's *z*-transformed Spearman correlations were computed between power and the behavioral variable step interval duration between the ipsilateral step at 0 and the preceding step. Encircled green clusters show that when the interval between the two steps was longer, beta power was higher. The same relationship is shown in the power average below (median split data; green, longest intervals; black, shortest intervals). The x denotes the average time of the 50% of all contralateral heel strikes with the longest intervals in green and the shortest intervals (i.e., occurring closer to the step at 0 s) in black.

Having confirmed the face validity of the data recoded during sitting, we tested whether beta modulation was stronger when the metronome was provided during stepping in place while sitting. Power modulation was computed for each condition as the difference between the maximum and minimum within 0–1 s and −0.5–0.5 s, respectively, so as to extract the peak and trough relative to the contralateral heel strike (Fig. 4). Beta modulation was significantly higher in the soundOn condition between 28 and 30 Hz. This modulation difference was driven by increased 28–30 Hz beta synchronization when the contralateral foot was resting on the ground [Fig. 7*A*; diff = 2.5%, *t*_(8)_ = 3. 05, *p* = 0.016, Hedges *g* = 0.30 (0.13, 0.68)], whereas the difference at the time of the trough was not significant [diff = −0.7%, *t*_(8)_ = −0.64, *p* = 0.542, Hedges *g* = −0.12 (−0.83, 0.20)].

### Auditory cueing-related differences in beta burst properties

The observed increased power peak in the soundOn condition may be due to a consistently increased amplitude of beta oscillations when the contralateral foot was resting on the ground or alternatively due to an increased occurrence rate of enhanced beta periods or bursts in some trials (which would also be expressed as higher amplitude in the average). The probability of 28–30 Hz beta bursts to occur was significantly higher in the soundOn condition (Fig. 7*B*). The median burst amplitude and burst duration did not differ significantly, indicating that the shape of the beta burst profile was not consistently different in the two conditions (burst amplitude: soundOn = 0.35, soundOff = 0.35, Wilcoxon signed rank test (*n* = 9), *p* = 0.570; burst duration: soundOn = 265 ms, soundOff = 255 ms, *t*_(8)_ = 0.5, *p* = 0.169).

### Within-subject correlations between power and behavior

To test whether beta modulation relates to the interval between two consecutive steps, we computed within-subjects correlations between trialwise (i.e., for each step) beta power and the step interval duration. We tested whether the Fisher's *z*-transformed correlations were significantly different from zero at the group level, separately in the soundOff and soundOn condition (Fig. 7*C*, left and right columns). The positive correlations (encircled green blobs) show that 20–30 Hz beta power was higher when the interval between the two steps was longer (see also the line plots below that depict the power average of the 50% longest and shortest interval durations in green and gray, respectively). If the contralateral heel strike preceded the current one (at time = 0 s) by >1 s (Fig. 7*C*, green x), then the beta rebound occurred earlier in both conditions. Interestingly, in the soundOn condition the beta rebound was not only higher when an interval was longer but it also decreased faster before the ipsilateral heel strike. No significant clusters were found when correlations with step-to-cue offsets were performed.

## Discussion

We found that during stepping in place, whether while sitting or standing, beta oscillations are modulated relative to the contralateral foot step cycle in the left and right STN in Parkinson's patients on levodopa. Importantly, beta band modulation was also observed between the STN during free walking in patients off levodopa, supporting the generalizability of our results to real gait and across drug states. Our results are in line with reports that STN beta power is modulated during repetitive tapping (Androulidakis et al., 2008; Joundi et al., 2013) and single limb movements (for review, see Brown, 2007), when beta also decreases and then rebounds, particularly for contralateral movements. However, these last reports bring in to question whether the rhythmic pattern of beta modulation observed here merely relates to the concatenation of the modulation seen with single limb movements, which would still be seen as conserved across different stepping-related paradigms. We do not argue against this possibility but rather point to some observations that might suggest that this core pattern is modified so that it becomes particularly functionally relevant in repetitive stepping. Rhythmic auditory cues, which can alleviate gait disturbances (Hausdorff et al., 2007; Arias and Cudeiro, 2008, 2010; Mazilu et al., 2015), assisted patients in reducing step timing variability and promoted power modulation in the beta band selectively. More precisely, when auditory cues were provided, the likelihood of beta bursts was increased after the contralateral heel strike. In addition, studies of single or repetitive upper limb movements report homogenous power changes over a broad beta band covering 13–30 Hz (Tan et al., 2013), whereas we found step-specific modulation to be most pronounced between 20 and 35 Hz during lower limb stepping movements.

### Is step-related modulation of high-beta oscillations primarily physiological?

We recorded from patients with Parkinson's disease, and as such the modulation of high-beta frequencies might be pathological rather than primarily physiological although we did not specifically investigate differences in beta modulation related to symptom severity across subjects. The latter necessitates larger sample sizes than in this study.

However, there are reasons for thinking that the modulation patterns shown here may reflect broadly similar modulation patterns in healthy subjects. First, patients performed the main stepping while sitting task on dopaminergic medication to minimize pathological activity linked to low levels of dopamine (Weinberger et al., 2006). Second, modulation patterns resembled those recorded with EEG electrodes over motor cortex in healthy subjects (Cheron et al., 2012; Seeber et al., 2014, 2015; Storzer et al., 2016). Within the somatotopic arrangement of the motor and premotor cortex, lower limbs are represented mesially (Godschalk et al., 1995) and coherence between these mesial cortical regions and the STN is particularly strong in the high beta band (Fogelson et al., 2006; Hirschmann et al., 2011; Litvak et al., 2011; Oswal et al., 2016), which supports the idea of a physiological role. Third, in hemiparkinsonian rats, dopaminergic lesions result in a specific increase of 25–40 Hz beta power during walking in the substantia nigra pars reticulata, which receives input from the STN (Avila et al., 2010). This activity is modulated relative to the contralateral paw movement similar as shown here (Brazhnik et al., 2014; Delaville et al., 2015).

### Does the degree of step-related modulation of high-beta oscillations relate to performance?

Within-subject correlations showed that the timing of the beta rebound was locked to the contralateral heel strike. Interestingly, in the soundOn condition the beta rebound was not only higher when an interval was longer but it also decreased faster before the ipsilateral heel strike. Immediately after each ipsilateral heel strike the contralateral foot is lifted. This faster beta decrease may thus support faster lifting of the contralateral foot, which may help to shorten the next interval to keep up with the rhythm. The increased modulation of beta power during long intervals may thus be an important contributor to the improved synchronization performance when auditory cues were provided. This resembles the increase in movement-related modulation of beta activity that can be observed after intake of antiparkinsonian medication, which generally enables patients to move faster (Doyle et al., 2005; Androulidakis et al., 2007).

The possible significance of step-cycle-locked modulation of high-beta oscillations is heightened by the fact that those patients, who found it harder to synchronize their steps to the rhythm when auditory cues were provided, tended to have more severe clinical gait impairments, in line with past findings (Plotnik and Hausdorff, 2008; Gilat et al., 2013). The absence of a similarly clear relationship in the soundOff condition can be attributed to the fact that some patients showed high behavioral variability although they did not score highly on the FOG-Q. This may be related to a general impairment in rhythm perception that could be unrelated to gait impairments and may be particularly severe in the absence of the metronome when the rhythm had to be extracted from the video alone.

Just as auditory cues can improve gait (Hausdorff et al., 2007; Arias and Cudeiro, 2008, 2010; Mazilu et al., 2015), we found that they improved patients' ability to match their steps to the heel strikes in the video, which was associated with an increased contralateral post-movement rebound in the upper beta frequency band. This average increase predominantly originated from an increased likelihood of individual beta bursts rather than a consistent increase in the degree of synchronization (i.e., amplitude) across all trials. It should be noted, though, that the peak amplitude of bursts may vary if different windows of interest had been chosen.

### What is the nature of the relationship between step-related modulation of high-beta oscillations and performance?

In patients suffering from freezing of gait, enhanced beta band synchronization was reported as a prominent feature both at rest (Toledo et al., 2014), and before and during freezing episodes (Singh et al., 2013; Shine et al., 2014; Scholten et al., 2016; Storzer et al., 2017). Exaggerated beta activity and lack of dynamic modulation of beta are considered to be correlates of upper limb motor impairment in Parkinson's disease (Androulidakis et al., 2007). In short, strong synchronization in the beta band and limited task-related modulation have been proposed to restrict local processing through rate coding and the dynamic configuration of neural assemblies on a finer spatiotemporal scale (Brittain and Brown, 2014). One hypothesis originating from the present results is that lack of dynamic modulation may also compromise local basal ganglia processing related to gait control.

Although we recorded from subthalamic electrodes, neurodegeneration in Parkinson's is not limited to the basal ganglia but also affects the pedunculopontine nucleus (PPN), other brainstem regions and cortico-subcortical processing (Alexander, 2004). Gait dysfunction could thus result from pathological changes outside the basal ganglia. The PPN as part of the mesencephalic locomotor region may be a key contributor to our observations. It has direct reciprocal connections to the STN as well with other basal ganglia nuclei, electrical stimulation elicits locomotion, and its neurons respond to auditory inputs with short latencies even in decerebrate animals (Reese et al., 1995; Mena-Segovia et al., 2004).

### Study limitations

Two more general caveats should also be highlighted. First, ours was a correlative study and so we cannot distinguish whether beta modulation is involved in the feedforward control of stepping; for example, by contributing to the estimation of interval timing as reported before (Kononowicz and van Rijn, 2015), or more indirectly involved, as a response to stepping-related sensory afferance or feedback-related error processing. The latter may have contributed to the observed association between better performance when the sound was present and higher modulation, as previous studies have shown that post-movement beta oscillations are more pronounced after correct versus erroneous movements (Tan et al., 2014, 2016b). Another possibility is that the reduced likelihood of post-movement beta oscillations in the soundOff condition was due to increased cognitive load (Fischer et al., 2016) when patients had to extract the heel strike timing solely from the visual information and struggled to stay on time.

Second, we should acknowledge that the DBS electrodes in our study may have also captured activity from surrounding structures given the small size of the STN, although the out-of-phase beta power dynamics between the two STN place an upper bound on the possibility of volume conduction. Moreover, LFP recordings could potentially also have been affected by microlesions caused by the electrode insertion procedure.

A further limitation of this study is that we recorded the auditory cueing condition only during stepping in place while sitting but not during unrestrained walking. However, the alternating pattern of modulation was very similar during unrestrained walking and freezing episodes can also be observed during stepping in place (Nantel et al., 2011) although no distinct freezing episodes were observed in our stepping task. Note that stepping on a foot pedal has also been used as a task for fMRI studies to investigate gait-related network dysfunctions in Parkinson's disease, although these studies displayed a first-person perspective virtual-reality corridor on a screen and did not constrain patients to step at a specific rhythm or speed (Shine et al., 2013a,b; Gilat et al., 2017).

### Implications for DBS

The present results suggest a link between rhythmic stepping performance and modulation of high-beta oscillations although we emphasize that we cannot infer any conclusions about a causal role of beta modulation from the current correlative data. The clear step-locked modulation pattern and its amplification with auditory cues raise the possibility that patients with gait problems may potentially benefit from temporally patterned left-right alternating DBS more than from continuous, uniform stimulation that would attenuate beta activity throughout the whole gait cycle. Instead, beta oscillations could be permitted at those points in the cycle at which they would naturally occur by briefly deactivating DBS; an assisted form of beta modulation, much in the same way as auditory cues seemed to redistribute beta bursts during stepping. Entrainment of motor cortical beta to auditory cues has recently been shown to be impaired in Parkinson's disease (te Woerd et al., 2017) and thus the benefit from auditory cueing alone will be limited. The hope is that alternating DBS patterns may turn out to provide a means to alleviate gait disturbances in those patients with impairments that are refractory to conventional DBS. This remains to be tested.

## References

[B1] Alexander GE (2004) Biology of Parkinson's disease: pathogenesis and pathophysiology of a multisystem neurodegenerative disorder. Dialogues Clin Neurosci 6:259–280. 22033559 10.31887/DCNS.2004.6.3/galexander PMC3181806

[B2] Androulidakis AG, Kühn AA, Chen CC, Blomstedt P, Kempf F, Kupsch A, Schneider GH, Doyle L, Dowsey-Limousin P, Hariz MI, Brown P (2007) Dopaminergic therapy promotes lateralized motor activity in the subthalamic area in Parkinson's disease. Brain 130:457–468. 10.1093/brain/awl358 17213215

[B3] Androulidakis AG, Brücke C, Kempf F, Kupsch A, Aziz T, Ashkan K, Kühn AA, Brown P (2008) Amplitude modulation of oscillatory activity in the subthalamic nucleus during movement. Eur J Neurosci 27:1277–1284. 10.1111/j.1460-9568.2008.06085.x 18312587

[B4] Anzak A, Tan H, Pogosyan A, Foltynie T, Limousin P, Zrinzo L, Hariz M, Ashkan K, Bogdanovic M, Green AL, Aziz T, Brown P (2012) Subthalamic nucleus activity optimizes maximal effort motor responses in Parkinson's disease. Brain 135:2766–2778. 10.1093/brain/aws183 22858550 PMC3437023

[B5] Arias P, Cudeiro J (2008) Effects of rhythmic sensory stimulation (auditory, visual) on gait in Parkinson's disease patients. Exp Brain Res 186:589–601. 10.1007/s00221-007-1263-y 18214453

[B6] Arias P, Cudeiro J (2010) Effect of rhythmic auditory stimulation on gait in parkinsonian patients with and without freezing of gait. PLoS One 5:e9675. 10.1371/journal.pone.0009675 20339591 PMC2842293

[B7] Arlotti M, Rosa M, Marceglia S, Barbieri S, Priori A (2016) The adaptive deep brain stimulation challenge. Parkinsonism Relat Disord 28:12–17. 10.1016/j.parkreldis.2016.03.020 27079257

[B8] Avila I, Parr-Brownlie LC, Brazhnik E, Castañeda E, Bergstrom DA, Walters JR (2010) Beta frequency synchronization in basal ganglia output during rest and walk in a hemiparkinsonian rat. Exp Neurol 221:307–319. 10.1016/j.expneurol.2009.11.016 19948166 PMC3384738

[B9] Baltadjieva R, Giladi N, Gruendlinger L, Peretz C, Hausdorff JM (2006) Marked alterations in the gait timing and rhythmicity of patients with *de novo* Parkinson's disease. Eur J Neurosci 24:1815–1820. 10.1111/j.1460-9568.2006.05033.x 17004944

[B10] Benjamini Y, Hochberg Y (1995) Controlling the false discovery rate: a practical and powerful approach to multiple testing. J R Stat Soc Series B Stat Methodol 57:289–300.

[B11] Bradford JC, Lukos JR, Ferris DP (2016) Electrocortical activity distinguishes between uphill and level walking in humans. J Neurophysiol 115:958–966. 10.1152/jn.00089.2015 26683062

[B12] Brazhnik E, Novikov N, McCoy AJ, Cruz AV, Walters JR (2014) Functional correlates of exaggerated oscillatory activity in basal ganglia output in hemiparkinsonian rats. Exp Neurol 261:563–577. 10.1016/j.expneurol.2014.07.010 25084518 PMC4318574

[B13] Brittain JS, Brown P (2014) Oscillations and the basal ganglia: motor control and beyond. Neuroimage 85:637–647. 10.1016/j.neuroimage.2013.05.084 23711535 PMC4813758

[B14] Brown P (2007) Abnormal oscillatory synchronisation in the motor system leads to impaired movement. Curr Opin Neurobiol 17:656–664. 10.1016/j.conb.2007.12.001 18221864

[B15] Cheron G, Duvinage M, De Saedeleer C, Castermans T, Bengoetxea A, Petieau M, Seetharaman K, Hoellinger T, Dan B, Dutoit T, Sylos Labini F, Lacquaniti F, Ivanenko Y (2012) From spinal central pattern generators to cortical network: integrated BCI for walking rehabilitation. Neural Plast 2012:375148. 10.1155/2012/375148 22272380 PMC3261492

[B16] Delaville C, McCoy AJ, Gerber CM, Cruz AV, Walters JR (2015) Subthalamic nucleus activity in the awake hemiparkinsonian rat: relationships with motor and cognitive networks. J Neurosci 35:6918–6930. 10.1523/JNEUROSCI.0587-15.2015 25926466 PMC4412903

[B17] Doyle LMF, Kühn AA, Hariz M, Kupsch A, Schneider GH, Brown P (2005) Levodopa-induced modulation of subthalamic beta oscillations during self-paced movements in patients with Parkinson's disease. Eur J Neurosci 21:1403–1412. 15813950 10.1111/j.1460-9568.2005.03969.x

[B18] Ebersbach G, Moreau C, Gandor F, Defebvre L, Devos D (2013) Clinical syndromes: parkinsonian gait. Mov Disord 28:1552–1559. 10.1002/mds.25675 24132843

[B19] Fasano A, Aquino CC, Krauss JK, Honey CR, Bloem BR (2015) Axial disability and deep brain stimulation in patients with Parkinson disease. Nat Rev Neurol 11:98–110. 10.1038/nrneurol.2014.252 25582445

[B20] Fischer P, Tan H, Pogosyan A, Brown P (2016) High post-movement parietal low-beta power during rhythmic tapping facilitates performance in a stop task. Eur J Neurosci 44:2202–2213. 10.1111/ejn.13328 27364852 PMC5014120

[B21] Fogelson N, Williams D, Tijssen M, van Bruggen G, Speelman H, Brown P (2006) Different functional loops between cerebral cortex and the subthalmic area in parkinson's disease. Cereb Cortex 16:64–75. 10.1093/cercor/bhi084 15829734

[B22] Fujioka T, Trainor LJ, Large EW, Ross B (2012) Internalized timing of isochronous sounds is represented in neuromagnetic beta oscillations. J Neurosci 32:1791–1802. 10.1523/JNEUROSCI.4107-11.2012 22302818 PMC6703342

[B23] Fujioka T, Ross B, Trainor LJ (2015) Beta-band oscillations represent auditory beat and its metrical hierarchy in perception and imagery. J Neurosci 35:15187–15198. 10.1523/JNEUROSCI.2397-15.2015 26558788 PMC6605356

[B24] Giladi N, Shabtai H, Simon ES, Biran S, Tal J, Korczyn AD (2000) Construction of freezing of gait questionnaire for patients with parkinsonism. Parkinsonism Relat Disord 6:165–170. 10.1016/S1353-8020(99)00062-0 10817956

[B25] Gilat M, Bell PT, Ehgoetz Martens KA, Georgiades MJ, Hall JM, Walton CC, Lewis SJG, Shine JM (2017) Dopamine depletion impairs gait automaticity by altering cortico-striatal and cerebellar processing in Parkinson's disease. Neuroimage 152:207–220. 10.1016/j.neuroimage.2017.02.073 28263926

[B26] Gilat M, Shine JM, Bolitho SJ, Matar E, Kamsma YPT, Naismith SL, Lewis SJG (2013) Variability of Stepping during a Virtual Reality Paradigm in Parkinson's Disease Patients with and without Freezing of Gait. PLoS One 8:1–6. 10.1371/journal.pone.0066718 PMC3689740 23805270

[B27] Godschalk M, Mitz AR, van Duin B, van der Burg H (1995) Somatotopy of monkey premotor cortex examined with microstimulation. Neurosci Res 23:269–279. 10.1016/0168-0102(95)00950-7 8545075

[B28] Hausdorff JM, Lowenthal J, Herman T, Gruendlinger L, Peretz C, Giladi N (2007) Rhythmic auditory stimulation modulates gait variability in Parkinson's disease. Eur J Neurosci 26:2369–2375. 10.1111/j.1460-9568.2007.05810.x 17953624

[B29] Hentschke H, Stüttgen MC (2011) Computation of measures of effect size for neuroscience data sets. Eur J Neurosci 34:1887–1894. 10.1111/j.1460-9568.2011.07902.x 22082031

[B30] Heremans E, Nieuwboer A, Vercruysse S (2013) Freezing of gait in Parkinson's disease: where are we now? Curr Neurol Neurosci Rep 13:350. 10.1007/s11910-013-0350-7 23625316

[B31] Hirschmann J, Özkurt TE, Butz M, Homburger M, Elben S, Hartmann CJ, Vesper J, Wojtecki L, Schnitzler A (2011) Distinct oscillatory STN-cortical loops revealed by simultaneous MEG and local field potential recordings in patients with Parkinson's disease. Neuroimage 55:1159–1168. 10.1016/j.neuroimage.2010.11.063 21122819

[B32] Holland PW, Welsch RE (1977) Robust regression using iteratively reweighted least-squares. Commun Stat Theory Methods 6:813–827. 10.1080/03610927708827533

[B33] Hollman JH, McDade EM, Petersen RC (2011) Normative spatiotemporal gait parameters in older adults. Gait Posture 34:111–118. 10.1016/j.gaitpost.2011.03.024 21531139 PMC3104090

[B34] Iversen JR, Repp BH, Patel AD (2009) Top-down control of rhythm perception modulates early auditory responses. Ann N Y Acad Sci 1169:58–73. 10.1111/j.1749-6632.2009.04579.x 19673755

[B35] Joundi RA, Brittain JS, Green AL, Aziz TZ, Brown P, Jenkinson N (2013) Persistent suppression of subthalamic beta-band activity during rhythmic finger tapping in Parkinson's disease. Clin Neurophysiol 124:565–573. 10.1016/j.clinph.2012.07.029 23085388

[B36] Kononowicz TW, van Rijn H (2015) Single trial beta oscillations index time estimation. Neuropsychologia 75:381–389. 10.1016/j.neuropsychologia.2015.06.014 26102187

[B37] Litvak V, Jha A, Eusebio A, Oostenveld R, Foltynie T, Limousin P, Zrinzo L, Hariz MI, Friston K, Brown P (2011) Resting oscillatory cortico-subthalamic connectivity in patients with Parkinson's disease. Brain 134:359–374. 10.1093/brain/awq332 21147836

[B38] Maris E, Oostenveld R (2007) Nonparametric statistical testing of EEG- and MEG-data. J Neurosci Methods 164:177–190. 10.1016/j.jneumeth.2007.03.024 17517438

[B39] Mazilu S, Blanke U, Dorfman M, Gazit E, Mirelman A, M. Hausdorff J, Tröster G (2015) A wearable assistant for gait training for Parkinson's disease with freezing of gait in out-of-the-lab environments. ACM Trans Interact Intell Syst 5:1–31. 10.1145/2701431

[B40] McIntosh GC, Brown SH, Rice RR, Thaut MH (1997) Rhythmic auditory-motor facilitation of gait patterns in patients with Parkinson's disease. J Neurol Neurosurg Psychiatry 62:22–26. 10.1136/jnnp.62.1.22 9010395 PMC486690

[B41] Meidahl AC, Tinkhauser G, Herz DM, Cagnan H, Debarros J, Brown P (2017) Adaptive deep brain stimulation for movement disorders: the long road to clinical therapy. Mov Disord 32:810–819. 10.1002/mds.27022 28597557 PMC5482397

[B42] Mena-Segovia J, Bolam JP, Magill PJ (2004) Pedunculopontine nucleus and basal ganglia: distant relatives or part of the same family? Trends Neurosci 27:585–588. 10.1016/j.tins.2004.07.009 15374668

[B43] Nantel J, de Solages C, Bronte-Stewart H (2011) Repetitive stepping in place identifies and measures freezing episodes in subjects with Parkinson's disease. Gait Posture 34:329–333. 21715166 10.1016/j.gaitpost.2011.05.020

[B44] Oostenveld R, Fries P, Maris E, Schoffelen JM (2011) FieldTrip: open source software for advanced analysis of MEG, EEG, and invasive electrophysiological data. Comput Intell Neurosci 2011:156869. 10.1155/2011/156869 21253357 PMC3021840

[B45] Oswal A, Beudel M, Zrinzo L, Limousin P, Hariz M, Foltynie T, Litvak V, Brown P (2016) Deep brain stimulation modulates synchrony within spatially and spectrally distinct resting state networks in Parkinson's disease. Brain 139:1482–1496. 10.1093/brain/aww048 27017189 PMC4845255

[B46] Pernet CR, Wilcox R, Rousselet GA (2013) Robust correlation analyses: false positive and power validation using a new open source MATLAB toolbox. Front Psychol 3:606. 10.3389/fpsyg.2012.00606 23335907 PMC3541537

[B47] Plotnik M, Hausdorff JM (2008) The role of gait rhythmicity and bilateral coordination of stepping in the pathophysiology of freezing of gait in Parkinson's disease. Mov Disord 23 (Suppl. 2):S444–S450. 18668626 10.1002/mds.21984

[B48] Reese NB, Garcia-Rill E, Skinner RD (1995) Auditory input to the pedunculopontine nucleus: II. Unit responses. Brain Res Bull 37:265–273. 10.1016/0361-9230(95)00001-U 7627569

[B49] Saleh M, Reimer J, Penn R, Ojakangas CL, Hatsopoulos NG (2010) Fast and slow oscillations in human primary motor cortex predict oncoming behaviorally relevant cues. Neuron 65:461–471. 10.1016/j.neuron.2010.02.001 20188651 PMC3199221

[B50] Scholten M, Govindan RB, Braun C, Bloem BR, Plewnia C, Krüger R, Gharabaghi A, Weiss D (2016) Cortical correlates of susceptibility to upper limb freezing in Parkinson's disease. Clin Neurophysiol 127:2386–2393. 10.1016/j.clinph.2016.01.028 27178857

[B51] Seeber M, Scherer R, Wagner J, Solis-Escalante T, Müller-Putz GR (2014) EEG beta suppression and low gamma modulation are different elements of human upright walking. Front Hum Neurosci 8:485. 10.3389/fnhum.2014.00485 25071515 PMC4086296

[B52] Seeber M, Scherer R, Wagner J, Solis-Escalante T, Müller-Putz GR (2015) High and low gamma EEG oscillations in central sensorimotor areas are conversely modulated during the human gait cycle. Neuroimage 112:318–326. 10.1016/j.neuroimage.2015.03.045 25818687

[B53] Shine JM, Matar E, Ward PB, Bolitho SJ, Gilat M, Pearson M, Naismith SL, Lewis SJ (2013a) Exploring the cortical and subcortical functional magnetic resonance imaging changes associated with freezing in Parkinson's disease. Brain 136:1204–1215. 10.1093/brain/awt049 23485851

[B54] Shine JM, Matar E, Ward PB, Frank MJ, Moustafa AA, Pearson M, Naismith SL, Lewis SJ (2013b) Freezing of gait in Parkinson's disease is associated with functional decoupling between the cognitive control network and the basal ganglia. Brain 136:3671–3681. 10.1093/brain/awt272 24142148

[B55] Shine JM, Handojoseno AM, Nguyen TN, Tran Y, Naismith SL, Nguyen H, Lewis SJ (2014) Abnormal patterns of theta frequency oscillations during the temporal evolution of freezing of gait in parkinson's disease. Clin Neurophysiol 125:569–576. 10.1016/j.clinph.2013.09.006 24099920

[B56] Singh A, Plate A, Kammermeier S, Mehrkens JH, Ilmberger J, Boetzel K (2013) Freezing of gait-related oscillatory activity in the human subthalamic nucleus. Basal Ganglia 3:25–32. 10.1016/j.baga.2012.10.002

[B57] Storzer L, Butz M, Hirschmann J, Abbasi O, Gratkowski M, Saupe D, Schnitzler A, Dalal SS (2016) Bicycling and walking are associated with different cortical oscillatory dynamics. Front Hum Neurosci 10:61. 10.3389/fnhum.2016.00061 26924977 PMC4759288

[B58] Storzer L, Butz M, Hirschmann J, Abbasi O, Gratkowski M, Saupe D, Vesper J, Dalal SS, Schnitzler A (2017) Bicycling suppresses abnormal beta synchrony in the parkinsonian basal ganglia. Ann Neurol 82:592–601. 10.1002/ana.25047 28892573

[B59] Tan H, Pogosyan A, Anzak A, Ashkan K, Bogdanovic M, Green AL, Aziz T, Foltynie T, Limousin P, Zrinzo L, Brown P (2013) Complementary roles of different oscillatory activities in the subthalamic nucleus in coding motor effort in parkinsonism. Exp Neurol 248:187–195. 10.1016/j.expneurol.2013.06.010 23778147 PMC3972632

[B60] Tan H, Zavala B, Pogosyan A, Ashkan K, Zrinzo L, Foltynie T, Limousin P, Brown P (2014) Human subthalamic nucleus in movement error detection and its evaluation during visuomotor adaptation. J Neurosci 34:16744–16754. 10.1523/JNEUROSCI.3414-14.2014 25505327 PMC4261099

[B61] Tan H, Pogosyan A, Ashkan K, Green AL, Aziz T, Foltynie T, Limousin P, Zrinzo L, Hariz M, Brown P (2016a) Decoding gripping force based on local field potentials recorded from subthalamic nucleus in humans. eLife 5:e19089. 10.7554/eLife.19089 27855780 PMC5148608

[B62] Tan H, Wade C, Brown P (2016b) Post-movement beta activity in sensorimotor cortex indexes confidence in the estimations from internal models. J Neurosci 36:1516–1528. 10.1523/JNEUROSCI.3204-15.2016 26843635 PMC4737767

[B63] te Woerd ES, Oostenveld R, de Lange FP, Praamstra P (2017) Impaired auditory-to-motor entrainment in Parkinson's disease. J Neurophysiol 117:1853–1864. 10.1152/jn.00547.2016 28179479 PMC5411464

[B64] Tinkhauser G, Pogosyan A, Little S, Beudel M, Herz DM, Tan H, Brown P (2017) The modulatory effect of adaptive deep brain stimulation on beta bursts in Parkinson's disease. Brain 140:1053–1067. 10.1093/brain/awx010 28334851 PMC5382944

[B65] Toledo JB, López-Azcárate J, Garcia-Garcia D, Guridi J, Valencia M, Artieda J, Obeso J, Alegre M, Rodriguez-Oroz M (2014) High beta activity in the subthalamic nucleus and freezing of gait in Parkinson's disease. Neurobiol Dis 64:60–65. 24361601 10.1016/j.nbd.2013.12.005

[B66] Tomlinson CL, Stowe R, Patel S, Rick C, Gray R, Clarke CE (2010) Systematic review of levodopa dose equivalency reporting in Parkinson's disease. Mov Disord 25:2649–2653. 10.1002/mds.23429 21069833

[B67] Weinberger M, Mahant N, Hutchison WD, Lozano AM, Moro E, Hodaie M, Lang AE, Dostrovsky JO (2006) Beta oscillatory activity in the subthalamic nucleus and its relation to dopaminergic response in Parkinson's disease. J Neurophysiol 96:3248–3256. 10.1152/jn.00697.2006 17005611

[B68] Williams DC (2011) Finite sample correction factors for several simple robust estimators of normal standard deviation. J Stat Comput Simul 81:1697–1702. 10.1080/00949655.2010.499516

